# Supplementary material for: Procurement practices and value for money in State Corporations in Kenya
Source: PLoS One. 2024 Jun 18;19(6):e0303879. doi: 10.1371/journal.pone.0303879 (PMC11185499; doi:10.1371/journal.pone.0303879)
Supplement: S1 File — (PDF) [file pone.0303879.s001.pdf]

Gender	Age	Qualification	Years_served	Age_of_the_company	Position	Planning
1.00	3.00	4.00	3.00	4.00	1	4.00
1.00	4.00	5.00	4.00	4.00	2	2.00
2.00	4.00	4.00	4.00	4.00	2	5.00
1.00	4.00	5.00	4.00	4.00	1	2.00
2.00	3.00	4.00	4.00	4.00	1	4.00
1.00	1.00	3.00	3.00	3.00	2	5.00
1.00	2.00	4.00	3.00	3.00	1	5.00
1.00	4.00	5.00	4.00	4.00	2	2.00
2.00	4.00	4.00	4.00	4.00	2	4.00
1.00	4.00	5.00	4.00	4.00	1	2.00
2.00	3.00	4.00	4.00	4.00	1	4.00
1.00	1.00	3.00	2.00	2.00	2	2.00
1.00	3.00	4.00	3.00	4.00	1	4.00
1.00	4.00	5.00	4.00	4.00	2	2.00
2.00	4.00	4.00	4.00	4.00	2	5.00
1.00	4.00	5.00	4.00	4.00	1	2.00
2.00	3.00	4.00	4.00	4.00	1	4.00
1.00	4.00	3.00	2.00	3.00	2	2.00
1.00	2.00	4.00	3.00	2.00	1	5.00
1.00	4.00	5.00	4.00	4.00	2	2.00
2.00	5.00	2.00	4.00	4.00	2	4.00
1.00	4.00	5.00	4.00	4.00	1	4.00
2.00	3.00	4.00	4.00	4.00	1	4.00
1.00	4.00	2.00	2.00	3.00	2	2.00
1.00	2.00	4.00	3.00	3.00	1	4.00
1.00	5.00	5.00	4.00	4.00	2	2.00
2.00	5.00	4.00	4.00	4.00	2	5.00
1.00	4.00	5.00	4.00	4.00	1	4.00
2.00	3.00	4.00	4.00	4.00	1	4.00
1.00	3.00	3.00	2.00	3.00	2	4.00
1.00	2.00	4.00	3.00	3.00	1	4.00
1.00	5.00	5.00	4.00	4.00	2	4.00
2.00	4.00	4.00	4.00	4.00	2	4.00
1.00	5.00	5.00	4.00	4.00	1	4.00
2.00	5.00	4.00	4.00	4.00	1	4.00
1.00	4.00	3.00	2.00	2.00	2	4.00
1.00	3.00	4.00	3.00	4.00	1	4.00
1.00	4.00	5.00	4.00	4.00	2	2.00
2.00	4.00	4.00	4.00	4.00	2	5.00
1.00	4.00	5.00	4.00	4.00	1	2.00
2.00	3.00	4.00	4.00	4.00	1	4.00
1.00	1.00	3.00	3.00	3.00	2	5.00
1.00	2.00	4.00	3.00	3.00	1	5.00
1.00	4.00	5.00	4.00	4.00	2	2.00
2.00	4.00	4.00	4.00	4.00	2	4.00
1.00	4.00	5.00	4.00	4.00	1	2.00

2.00	3.00	4.00	4.00	4.00	1	4.00
1.00	1.00	3.00	2.00	2.00	2	2.00
1.00	3.00	4.00	3.00	4.00	1	4.00
1.00	4.00	5.00	4.00	4.00	2	2.00
2.00	4.00	4.00	4.00	4.00	2	5.00
1.00	4.00	5.00	4.00	4.00	1	2.00
2.00	3.00	4.00	4.00	4.00	1	4.00
1.00	4.00	3.00	2.00	3.00	2	2.00
1.00	2.00	4.00	3.00	2.00	1	5.00
1.00	4.00	5.00	4.00	4.00	2	2.00
2.00	5.00	2.00	4.00	4.00	2	4.00
1.00	4.00	5.00	4.00	4.00	1	4.00
2.00	3.00	4.00	4.00	4.00	1	4.00
1.00	4.00	2.00	2.00	3.00	2	2.00
1.00	2.00	4.00	3.00	3.00	1	4.00
1.00	5.00	5.00	4.00	4.00	2	2.00
2.00	5.00	4.00	4.00	4.00	2	5.00
1.00	4.00	5.00	4.00	4.00	1	4.00
2.00	3.00	4.00	4.00	4.00	1	4.00
1.00	3.00	3.00	2.00	3.00	2	4.00
1.00	2.00	4.00	3.00	3.00	1	4.00
1.00	5.00	5.00	4.00	4.00	2	4.00
2.00	4.00	4.00	4.00	4.00	2	4.00
1.00	5.00	5.00	4.00	4.00	1	4.00
2.00	5.00	4.00	4.00	4.00	1	4.00
1.00	4.00	3.00	2.00	2.00	2	4.00
1.00	3.00	4.00	3.00	4.00	1	4.00
1.00	4.00	5.00	4.00	4.00	2	2.00
2.00	4.00	4.00	4.00	4.00	2	5.00
1.00	4.00	5.00	4.00	4.00	1	2.00
2.00	3.00	4.00	4.00	4.00	1	4.00
1.00	1.00	3.00	3.00	3.00	2	5.00
1.00	2.00	4.00	3.00	3.00	1	5.00
1.00	4.00	5.00	4.00	4.00	2	2.00
2.00	4.00	4.00	4.00	4.00	2	4.00
1.00	4.00	5.00	4.00	4.00	1	2.00
2.00	3.00	4.00	4.00	4.00	1	4.00
1.00	1.00	3.00	2.00	2.00	2	2.00
1.00	3.00	4.00	3.00	4.00	1	4.00
1.00	4.00	5.00	4.00	4.00	2	2.00
2.00	4.00	4.00	4.00	4.00	2	5.00

Planning2	Planning3	Planning4	Planning5	Planning6	Planning7	Planning8	Sourcing2	Sourcing3
2.00	4.00	4.00	4.00	4.00	3.00	3.00	2.00	2.00
2.00	4.00	4.00	4.00	2.00	4.00	3.00	4.00	4.00
4.00	4.00	3.00	2.00	4.00	4.00	4.00	4.00	4.00
4.00	2.00	3.00	4.00	4.00	4.00	4.00	2.00	2.00
1.00	4.00	5.00	4.00	4.00	2.00	4.00	2.00	2.00
4.00	4.00	2.00	2.00	4.00	5.00	5.00	5.00	5.00
4.00	4.00	3.00	2.00	4.00	5.00	5.00	2.00	5.00
4.00	2.00	4.00	5.00	4.00	4.00	3.00	2.00	3.00
2.00	4.00	4.00	4.00	4.00	5.00	2.00	4.00	5.00
4.00	2.00	4.00	3.00	3.00	4.00	2.00	4.00	4.00
2.00	4.00	4.00	2.00	2.00	2.00	3.00	2.00	2.00
4.00	4.00	4.00	3.00	3.00	5.00	4.00	2.00	5.00
5.00	5.00	4.00	4.00	2.00	2.00	2.00	2.00	2.00
4.00	4.00	4.00	3.00	4.00	4.00	2.00	4.00	3.00
4.00	3.00	2.00	2.00	1.00	3.00	2.00	4.00	4.00
4.00	2.00	5.00	5.00	1.00	2.00	4.00	2.00	2.00
1.00	4.00	5.00	2.00	5.00	4.00	4.00	3.00	4.00
1.00	5.00	5.00	4.00	2.00	5.00	3.00	5.00	5.00
4.00	3.00	2.00	2.00	3.00	5.00	4.00	5.00	5.00
4.00	2.00	3.00	3.00	3.00	4.00	2.00	3.00	3.00
3.00	4.00	5.00	4.00	4.00	5.00	4.00	4.00	4.00
2.00	3.00	4.00	5.00	3.00	4.00	2.00	4.00	4.00
5.00	5.00	4.00	4.00	2.00	2.00	2.00	2.00	2.00
2.00	4.00	4.00	4.00	3.00	5.00	2.00	5.00	5.00
2.00	4.00	2.00	4.00	4.00	2.00	2.00	4.00	4.00
4.00	3.00	5.00	4.00	1.00	4.00	4.00	2.00	3.00
1.00	5.00	5.00	2.00	2.00	3.00	2.00	5.00	5.00
2.00	4.00	4.00	4.00	2.00	2.00	2.00	4.00	4.00
4.00	3.00	2.00	5.00	1.00	4.00	4.00	4.00	4.00
1.00	2.00	4.00	5.00	4.00	4.00	4.00	5.00	5.00
2.00	4.00	4.00	2.00	4.00	4.00	2.00	4.00	4.00
5.00	4.00	4.00	2.00	5.00	4.00	4.00	4.00	4.00
2.00	4.00	4.00	2.00	2.00	4.00	4.00	4.00	4.00
3.00	4.00	4.00	2.00	2.00	4.00	2.00	4.00	4.00
4.00	4.00	4.00	4.00	2.00	4.00	2.00	4.00	4.00
2.00	4.00	4.00	4.00	2.00	2.00	2.00	4.00	4.00
5.00	4.00	4.00	4.00	2.00	3.00	3.00	2.00	2.00
2.00	4.00	4.00	4.00	4.00	4.00	3.00	4.00	4.00
4.00	4.00	3.00	2.00	4.00	4.00	4.00	4.00	4.00
4.00	2.00	3.00	4.00	4.00	4.00	4.00	2.00	2.00
1.00	4.00	5.00	4.00	4.00	2.00	4.00	2.00	2.00
4.00	4.00	2.00	2.00	4.00	5.00	5.00	5.00	5.00
4.00	4.00	3.00	2.00	4.00	5.00	5.00	2.00	5.00
4.00	2.00	4.00	5.00	4.00	4.00	3.00	2.00	3.00
2.00	4.00	4.00	4.00	4.00	5.00	2.00	4.00	3.00
4.00	2.00	4.00	3.00	3.00	4.00	2.00	4.00	4.00

2.00	4.00	4.00	2.00	2.00	2.00	3.00	2.00	2.00
2.00	4.00	4.00	3.00	3.00	5.00	4.00	2.00	5.00
2.00	5.00	4.00	4.00	2.00	2.00	2.00	2.00	2.00
2.00	4.00	4.00	3.00	4.00	4.00	4.00	4.00	3.00
4.00	3.00	2.00	2.00	1.00	3.00	4.00	4.00	4.00
4.00	2.00	5.00	5.00	1.00	2.00	4.00	2.00	4.00
1.00	4.00	5.00	2.00	2.00	4.00	4.00	3.00	4.00
1.00	5.00	5.00	4.00	5.00	5.00	3.00	5.00	5.00
4.00	3.00	2.00	2.00	3.00	5.00	4.00	5.00	5.00
4.00	2.00	3.00	3.00	3.00	4.00	2.00	3.00	3.00
3.00	4.00	5.00	4.00	4.00	5.00	4.00	4.00	4.00
2.00	3.00	4.00	5.00	3.00	4.00	4.00	4.00	4.00
4.00	5.00	4.00	4.00	2.00	2.00	2.00	2.00	4.00
4.00	4.00	4.00	4.00	3.00	5.00	2.00	5.00	5.00
2.00	4.00	2.00	4.00	2.00	2.00	4.00	4.00	4.00
4.00	3.00	5.00	4.00	1.00	4.00	4.00	2.00	3.00
1.00	5.00	5.00	2.00	4.00	3.00	2.00	5.00	5.00
2.00	4.00	4.00	4.00	2.00	2.00	4.00	4.00	4.00
4.00	3.00	2.00	5.00	1.00	4.00	4.00	4.00	4.00
3.00	2.00	4.00	5.00	4.00	4.00	4.00	5.00	5.00
5.00	4.00	4.00	2.00	2.00	4.00	4.00	4.00	4.00
2.00	4.00	4.00	2.00	4.00	4.00	4.00	4.00	4.00
2.00	4.00	4.00	2.00	5.00	4.00	4.00	4.00	4.00
4.00	4.00	4.00	2.00	5.00	4.00	4.00	4.00	4.00
2.00	4.00	4.00	4.00	2.00	4.00	4.00	4.00	4.00
2.00	4.00	4.00	4.00	2.00	2.00	2.00	4.00	4.00
4.00	4.00	4.00	4.00	2.00	3.00	3.00	2.00	2.00
2.00	4.00	4.00	4.00	2.00	4.00	3.00	4.00	4.00
4.00	4.00	3.00	2.00	4.00	4.00	4.00	4.00	4.00
4.00	2.00	3.00	4.00	4.00	4.00	4.00	2.00	2.00
1.00	4.00	5.00	4.00	4.00	2.00	4.00	2.00	2.00
4.00	4.00	2.00	2.00	4.00	5.00	5.00	5.00	5.00
4.00	4.00	3.00	2.00	4.00	5.00	5.00	2.00	5.00
4.00	2.00	4.00	5.00	4.00	4.00	3.00	2.00	3.00
2.00	4.00	4.00	4.00	4.00	5.00	2.00	4.00	3.00
4.00	2.00	4.00	3.00	3.00	4.00	2.00	4.00	4.00
2.00	4.00	4.00	2.00	2.00	2.00	3.00	2.00	2.00
2.00	4.00	4.00	3.00	3.00	5.00	4.00	2.00	5.00
2.00	5.00	4.00	4.00	2.00	2.00	2.00	2.00	2.00
5.00	4.00	4.00	3.00	2.00	4.00	2.00	4.00	3.00
4.00	3.00	2.00	2.00	1.00	3.00	2.00	4.00	4.00

Sourcing4	Sourcing5	Sourcing6	Sourcing7	Sourcing8	Sourcing9	Sourcing10	Sourcing11	Sourcing12
4.00	4.00	4.00	2.00	4.00	4.00	2.00	2.00	4.00
2.00	4.00	5.00	2.00	3.00	2.00	2.00	2.00	4.00
2.00	2.00	4.00	4.00	2.00	4.00	2.00	2.00	5.00
2.00	3.00	5.00	4.00	4.00	4.00	4.00	4.00	5.00
3.00	5.00	5.00	3.00	4.00	4.00	4.00	4.00	1.00
3.00	4.00	4.00	4.00	2.00	4.00	4.00	2.00	4.00
4.00	2.00	4.00	4.00	2.00	2.00	3.00	3.00	4.00
3.00	3.00	3.00	2.00	2.00	5.00	2.00	4.00	2.00
4.00	3.00	4.00	2.00	3.00	4.00	4.00	4.00	4.00
3.00	5.00	5.00	4.00	4.00	5.00	5.00	3.00	4.00
4.00	4.00	4.00	2.00	4.00	4.00	4.00	2.00	2.00
4.00	2.00	3.00	2.00	3.00	3.00	3.00	3.00	3.00
4.00	4.00	4.00	2.00	4.00	4.00	4.00	2.00	2.00
4.00	4.00	5.00	2.00	3.00	2.00	2.00	2.00	4.00
2.00	2.00	2.00	4.00	2.00	4.00	4.00	4.00	1.00
4.00	3.00	2.00	3.00	2.00	3.00	3.00	4.00	4.00
5.00	5.00	5.00	3.00	4.00	5.00	5.00	3.00	3.00
5.00	3.00	4.00	4.00	4.00	3.00	3.00	3.00	4.00
2.00	2.00	2.00	2.00	2.00	3.00	3.00	4.00	4.00
4.00	3.00	5.00	4.00	2.00	5.00	5.00	3.00	4.00
5.00	5.00	5.00	3.00	3.00	4.00	4.00	4.00	3.00
4.00	4.00	4.00	2.00	2.00	5.00	5.00	3.00	4.00
4.00	4.00	4.00	2.00	3.00	4.00	4.00	2.00	2.00
4.00	2.00	5.00	2.00	4.00	3.00	3.00	3.00	4.00
4.00	4.00	4.00	4.00	4.00	4.00	4.00	4.00	2.00
4.00	4.00	4.00	4.00	4.00	4.00	4.00	4.00	4.00
4.00	4.00	4.00	4.00	4.00	4.00	4.00	4.00	4.00
4.00	4.00	4.00	4.00	4.00	4.00	4.00	4.00	2.00
4.00	4.00	4.00	4.00	4.00	4.00	4.00	4.00	4.00
4.00	4.00	4.00	4.00	4.00	4.00	4.00	4.00	4.00
4.00	4.00	4.00	4.00	4.00	4.00	4.00	4.00	2.00
2.00	2.00	2.00	2.00	2.00	2.00	2.00	2.00	4.00
2.00	2.00	2.00	2.00	2.00	2.00	2.00	2.00	4.00
4.00	4.00	4.00	4.00	4.00	4.00	4.00	4.00	2.00
4.00	4.00	4.00	4.00	4.00	4.00	4.00	4.00	2.00
2.00	2.00	2.00	2.00	2.00	2.00	2.00	2.00	4.00
4.00	4.00	4.00	2.00	4.00	4.00	2.00	2.00	2.00
2.00	2.00	5.00	2.00	3.00	2.00	2.00	2.00	2.00
2.00	2.00	2.00	4.00	2.00	4.00	2.00	2.00	1.00
2.00	3.00	5.00	4.00	4.00	4.00	4.00	4.00	1.00
3.00	5.00	5.00	3.00	4.00	4.00	4.00	4.00	5.00
3.00	2.00	2.00	4.00	2.00	4.00	4.00	2.00	2.00
4.00	2.00	2.00	4.00	2.00	2.00	3.00	3.00	4.00
3.00	3.00	3.00	2.00	2.00	5.00	2.00	4.00	2.00
4.00	3.00	4.00	2.00	3.00	4.00	4.00	4.00	4.00
3.00	5.00	5.00	4.00	4.00	5.00	5.00	3.00	4.00

4.00	4.00	4.00	2.00	4.00	4.00	4.00	2.00	2.00
4.00	2.00	3.00	2.00	3.00	3.00	3.00	3.00	3.00
4.00	4.00	4.00	2.00	4.00	4.00	4.00	2.00	4.00
4.00	2.00	5.00	2.00	3.00	2.00	2.00	2.00	2.00
2.00	2.00	2.00	4.00	2.00	4.00	4.00	4.00	1.00
4.00	3.00	4.00	3.00	2.00	3.00	3.00	4.00	4.00
5.00	5.00	5.00	3.00	4.00	5.00	5.00	3.00	2.00
5.00	3.00	4.00	4.00	4.00	3.00	3.00	3.00	2.00
2.00	2.00	2.00	2.00	2.00	3.00	3.00	4.00	4.00
4.00	3.00	5.00	4.00	2.00	5.00	5.00	3.00	4.00
5.00	5.00	5.00	3.00	3.00	4.00	4.00	4.00	3.00
4.00	4.00	4.00	2.00	2.00	5.00	5.00	3.00	2.00
4.00	4.00	4.00	4.00	3.00	4.00	4.00	2.00	4.00
4.00	4.00	5.00	4.00	4.00	3.00	3.00	3.00	4.00
4.00	4.00	4.00	4.00	4.00	4.00	4.00	4.00	2.00
4.00	4.00	4.00	4.00	4.00	4.00	4.00	4.00	4.00
4.00	4.00	4.00	4.00	4.00	4.00	4.00	4.00	4.00
4.00	4.00	4.00	4.00	4.00	4.00	4.00	4.00	2.00
4.00	4.00	4.00	4.00	4.00	4.00	4.00	4.00	4.00
4.00	4.00	4.00	4.00	4.00	4.00	4.00	4.00	4.00
4.00	4.00	4.00	4.00	4.00	4.00	4.00	4.00	4.00
2.00	2.00	2.00	4.00	2.00	2.00	2.00	2.00	4.00
2.00	2.00	2.00	2.00	2.00	2.00	2.00	2.00	4.00
4.00	4.00	4.00	4.00	4.00	4.00	4.00	4.00	4.00
4.00	4.00	4.00	4.00	4.00	4.00	4.00	4.00	4.00
2.00	2.00	2.00	2.00	2.00	2.00	2.00	2.00	4.00
4.00	4.00	4.00	4.00	4.00	4.00	2.00	2.00	2.00
2.00	2.00	5.00	2.00	3.00	2.00	2.00	2.00	4.00
2.00	2.00	2.00	4.00	2.00	4.00	2.00	2.00	1.00
2.00	3.00	5.00	4.00	4.00	4.00	4.00	4.00	5.00
3.00	5.00	5.00	3.00	4.00	4.00	4.00	4.00	5.00
3.00	2.00	2.00	4.00	2.00	4.00	4.00	2.00	4.00
4.00	2.00	2.00	4.00	2.00	2.00	3.00	3.00	4.00
3.00	3.00	3.00	2.00	2.00	5.00	2.00	4.00	3.00
4.00	3.00	4.00	4.00	3.00	4.00	4.00	4.00	4.00
3.00	5.00	5.00	4.00	4.00	5.00	5.00	3.00	4.00
4.00	4.00	4.00	4.00	4.00	4.00	4.00	2.00	2.00
4.00	2.00	3.00	2.00	3.00	3.00	3.00	3.00	3.00
4.00	4.00	4.00	4.00	4.00	4.00	4.00	2.00	3.00
4.00	2.00	5.00	2.00	3.00	2.00	2.00	2.00	4.00
2.00	2.00	2.00	4.00	2.00	4.00	4.00	4.00	3.00

Manageme	Manageme	Manageme	Manageme	Manageme	Manageme	Manageme	Manageme	Manageme
4.00	4.00	4.00	4.00	3.00	4.00	2.00	4.00	4.00
4.00	4.00	4.00	4.00	4.00	4.00	4.00	2.00	4.00
4.00	2.00	2.00	2.00	4.00	2.00	4.00	2.00	4.00
1.00	2.00	2.00	2.00	2.00	2.00	4.00	2.00	4.00
1.00	3.00	5.00	3.00	4.00	2.00	4.00	2.00	3.00
2.00	3.00	3.00	3.00	5.00	5.00	5.00	4.00	2.00
2.00	2.00	2.00	2.00	3.00	2.00	5.00	4.00	2.00
4.00	2.00	2.00	2.00	2.00	2.00	3.00	3.00	4.00
4.00	4.00	4.00	4.00	3.00	4.00	4.00	1.00	4.00
3.00	2.00	2.00	2.00	2.00	2.00	3.00	4.00	4.00
2.00	4.00	4.00	4.00	4.00	2.00	2.00	4.00	2.00
3.00	4.00	4.00	4.00	4.00	4.00	5.00	3.00	3.00
4.00	4.00	4.00	4.00	3.00	4.00	2.00	4.00	4.00
2.00	4.00	4.00	4.00	4.00	4.00	4.00	2.00	3.00
1.00	2.00	2.00	2.00	3.00	2.00	4.00	2.00	2.00
4.00	2.00	2.00	2.00	2.00	4.00	4.00	2.00	1.00
3.00	3.00	5.00	3.00	4.00	5.00	3.00	4.00	4.00
2.00	3.00	3.00	2.00	5.00	3.00	5.00	4.00	4.00
2.00	2.00	2.00	2.00	4.00	2.00	5.00	4.00	4.00
4.00	2.00	2.00	2.00	2.00	4.00	3.00	3.00	1.00
4.00	3.00	5.00	3.00	4.00	4.00	4.00	1.00	3.00
3.00	4.00	4.00	2.00	4.00	2.00	3.00	5.00	4.00
2.00	4.00	4.00	2.00	3.00	4.00	2.00	4.00	2.00
3.00	4.00	4.00	4.00	4.00	4.00	5.00	5.00	3.00
2.00	4.00	4.00	4.00	4.00	4.00	2.00	4.00	4.00
2.00	1.00	1.00	1.00	2.00	1.00	4.00	4.00	4.00
2.00	4.00	4.00	4.00	4.00	4.00	2.00	4.00	4.00
4.00	4.00	4.00	4.00	4.00	4.00	4.00	4.00	4.00
5.00	4.00	4.00	4.00	4.00	4.00	2.00	4.00	4.00
4.00	4.00	4.00	4.00	4.00	4.00	4.00	4.00	4.00
2.00	3.00	4.00	3.00	4.00	3.00	2.00	2.00	4.00
4.00	5.00	3.00	2.00	1.00	2.00	4.00	2.00	4.00
4.00	5.00	2.00	1.00	2.00	3.00	4.00	5.00	2.00
2.00	4.00	4.00	4.00	4.00	4.00	2.00	4.00	4.00
2.00	1.00	1.00	1.00	2.00	1.00	4.00	4.00	4.00
4.00	4.00	4.00	4.00	4.00	4.00	2.00	4.00	2.00
1.00	4.00	4.00	4.00	3.00	4.00	2.00	4.00	4.00
1.00	4.00	4.00	4.00	4.00	4.00	4.00	2.00	4.00
4.00	2.00	2.00	2.00	4.00	2.00	4.00	2.00	4.00
4.00	5.00	2.00	2.00	2.00	4.00	4.00	2.00	4.00
4.00	3.00	3.00	3.00	4.00	2.00	4.00	2.00	4.00
2.00	3.00	3.00	3.00	5.00	3.00	5.00	4.00	2.00
2.00	2.00	2.00	2.00	3.00	2.00	5.00	4.00	2.00
4.00	2.00	2.00	2.00	2.00	2.00	3.00	4.00	4.00
4.00	4.00	4.00	4.00	3.00	4.00	4.00	1.00	2.00
3.00	2.00	2.00	2.00	2.00	2.00	3.00	4.00	4.00

2.00	4.00	4.00	4.00	4.00	2.00	2.00	4.00	4.00
3.00	4.00	4.00	4.00	4.00	4.00	5.00	4.00	4.00
2.00	4.00	4.00	4.00	3.00	4.00	2.00	4.00	2.00
2.00	4.00	4.00	4.00	4.00	4.00	4.00	2.00	3.00
1.00	2.00	2.00	2.00	3.00	2.00	4.00	2.00	2.00
1.00	2.00	2.00	2.00	2.00	2.00	4.00	2.00	1.00
3.00	3.00	3.00	3.00	4.00	5.00	3.00	4.00	4.00
2.00	3.00	3.00	3.00	5.00	3.00	5.00	4.00	4.00
2.00	2.00	2.00	2.00	4.00	2.00	5.00	4.00	2.00
4.00	2.00	2.00	2.00	2.00	2.00	3.00	3.00	1.00
4.00	3.00	3.00	3.00	4.00	4.00	4.00	1.00	5.00
3.00	4.00	4.00	4.00	4.00	2.00	3.00	3.00	2.00
2.00	4.00	4.00	4.00	3.00	4.00	2.00	4.00	2.00
3.00	4.00	4.00	4.00	4.00	4.00	5.00	3.00	5.00
4.00	4.00	4.00	4.00	4.00	4.00	2.00	4.00	4.00
4.00	1.00	1.00	1.00	2.00	1.00	4.00	4.00	4.00
4.00	4.00	4.00	4.00	4.00	4.00	2.00	4.00	4.00
4.00	4.00	4.00	4.00	4.00	4.00	4.00	4.00	4.00
4.00	4.00	4.00	4.00	4.00	4.00	2.00	4.00	4.00
4.00	4.00	4.00	4.00	4.00	4.00	4.00	4.00	4.00
4.00	3.00	4.00	3.00	4.00	3.00	2.00	2.00	4.00
4.00	2.00	3.00	2.00	1.00	2.00	4.00	2.00	4.00
4.00	1.00	2.00	1.00	2.00	3.00	4.00	3.00	2.00
2.00	4.00	4.00	4.00	4.00	4.00	2.00	4.00	4.00
4.00	1.00	1.00	1.00	2.00	1.00	4.00	4.00	2.00
4.00	4.00	4.00	4.00	4.00	4.00	2.00	4.00	2.00
1.00	4.00	4.00	4.00	3.00	4.00	2.00	4.00	2.00
5.00	4.00	4.00	4.00	4.00	4.00	4.00	2.00	4.00
5.00	2.00	2.00	2.00	4.00	2.00	4.00	2.00	4.00
5.00	2.00	2.00	2.00	2.00	2.00	4.00	2.00	4.00
5.00	3.00	3.00	3.00	4.00	2.00	4.00	2.00	5.00
2.00	3.00	3.00	3.00	5.00	3.00	5.00	4.00	2.00
2.00	2.00	2.00	2.00	3.00	2.00	5.00	4.00	4.00
4.00	2.00	2.00	2.00	2.00	2.00	3.00	3.00	4.00
4.00	4.00	4.00	4.00	3.00	4.00	4.00	1.00	2.00
3.00	2.00	2.00	2.00	2.00	2.00	3.00	3.00	4.00
2.00	4.00	4.00	4.00	4.00	2.00	2.00	4.00	4.00
3.00	4.00	4.00	4.00	4.00	4.00	5.00	3.00	5.00
4.00	4.00	4.00	4.00	3.00	4.00	2.00	4.00	4.00
4.00	4.00	4.00	4.00	4.00	4.00	4.00	2.00	3.00
5.00	2.00	2.00	2.00	3.00	2.00	4.00	2.00	2.00

Manageme	Manageme	eprocurem	eprocurem	eprocurem	eprocurem	eprocurem	eprocurem	eprocurem
2.00	2.00	2.00	3.00	2.00	2.00	2.00	2.00	2.00
2.00	4.00	4.00	4.00	2.00	4.00	2.00	4.00	4.00
4.00	2.00	4.00	4.00	1.00	1.00	1.00	3.00	4.00
3.00	2.00	4.00	4.00	1.00	4.00	4.00	4.00	4.00
3.00	2.00	3.00	4.00	1.00	1.00	1.00	1.00	1.00
4.00	1.00	3.00	2.00	4.00	4.00	4.00	5.00	3.00
4.00	3.00	3.00	2.00	4.00	4.00	4.00	5.00	5.00
4.00	4.00	5.00	2.00	4.00	3.00	4.00	3.00	3.00
2.00	2.00	2.00	3.00	3.00	4.00	2.00	4.00	4.00
4.00	4.00	5.00	2.00	4.00	3.00	4.00	4.00	3.00
2.00	2.00	2.00	4.00	2.00	2.00	2.00	2.00	2.00
4.00	4.00	4.00	3.00	4.00	3.00	4.00	5.00	3.00
2.00	2.00	2.00	3.00	2.00	2.00	2.00	2.00	2.00
2.00	4.00	4.00	3.00	2.00	2.00	2.00	4.00	2.00
4.00	1.00	3.00	4.00	1.00	4.00	1.00	4.00	1.00
4.00	5.00	5.00	2.00	1.00	1.00	1.00	1.00	4.00
2.00	3.00	3.00	4.00	4.00	3.00	4.00	3.00	4.00
3.00	5.00	5.00	4.00	4.00	4.00	4.00	5.00	5.00
4.00	3.00	3.00	2.00	4.00	4.00	4.00	5.00	5.00
4.00	5.00	5.00	4.00	4.00	3.00	4.00	3.00	3.00
3.00	3.00	2.00	3.00	5.00	4.00	2.00	4.00	4.00
2.00	2.00	2.00	2.00	4.00	3.00	4.00	3.00	3.00
2.00	2.00	2.00	3.00	2.00	2.00	2.00	2.00	2.00
2.00	4.00	4.00	3.00	4.00	3.00	4.00	5.00	5.00
2.00	4.00	4.00	4.00	2.00	2.00	2.00	4.00	4.00
4.00	4.00	4.00	4.00	2.00	2.00	2.00	4.00	4.00
2.00	4.00	4.00	4.00	2.00	2.00	2.00	4.00	4.00
2.00	4.00	4.00	4.00	2.00	2.00	2.00	4.00	4.00
4.00	4.00	4.00	4.00	2.00	2.00	2.00	4.00	4.00
2.00	4.00	4.00	4.00	2.00	2.00	2.00	4.00	4.00
4.00	4.00	4.00	4.00	2.00	2.00	2.00	4.00	4.00
2.00	4.00	4.00	4.00	2.00	2.00	2.00	4.00	4.00
4.00	2.00	2.00	2.00	3.00	4.00	3.00	2.00	2.00
4.00	2.00	2.00	2.00	5.00	4.00	3.00	2.00	2.00
4.00	2.00	2.00	2.00	3.00	4.00	2.00	2.00	2.00
4.00	2.00	2.00	2.00	5.00	4.00	3.00	2.00	2.00
4.00	2.00	2.00	3.00	2.00	2.00	2.00	2.00	2.00
4.00	4.00	4.00	3.00	2.00	4.00	2.00	4.00	4.00
4.00	3.00	4.00	4.00	1.00	1.00	1.00	3.00	4.00
3.00	1.00	4.00	4.00	1.00	4.00	4.00	4.00	4.00
5.00	1.00	3.00	4.00	1.00	1.00	1.00	1.00	1.00
4.00	3.00	3.00	2.00	4.00	4.00	4.00	5.00	3.00
4.00	1.00	3.00	2.00	4.00	4.00	4.00	5.00	5.00
4.00	4.00	5.00	2.00	4.00	5.00	4.00	3.00	3.00
2.00	2.00	2.00	3.00	3.00	4.00	3.00	4.00	4.00
4.00	4.00	5.00	2.00	4.00	2.00	4.00	4.00	4.00

2.00	2.00	2.00	4.00	2.00	2.00	2.00	2.00	2.00
4.00	4.00	4.00	3.00	4.00	2.00	4.00	5.00	3.00
2.00	2.00	2.00	3.00	2.00	2.00	2.00	2.00	2.00
2.00	4.00	4.00	3.00	2.00	2.00	2.00	4.00	2.00
4.00	3.00	3.00	4.00	1.00	4.00	1.00	4.00	1.00
4.00	5.00	5.00	2.00	1.00	1.00	1.00	1.00	4.00
2.00	3.00	3.00	4.00	4.00	2.00	4.00	3.00	3.00
3.00	5.00	5.00	4.00	4.00	4.00	4.00	5.00	5.00
4.00	3.00	3.00	2.00	4.00	4.00	4.00	5.00	5.00
4.00	5.00	5.00	4.00	4.00	2.00	4.00	3.00	4.00
3.00	2.00	2.00	1.00	3.00	4.00	3.00	4.00	4.00
2.00	2.00	2.00	2.00	4.00	2.00	4.00	3.00	3.00
2.00	2.00	2.00	3.00	2.00	2.00	2.00	2.00	2.00
2.00	4.00	4.00	3.00	4.00	3.00	4.00	5.00	5.00
4.00	4.00	4.00	4.00	2.00	2.00	2.00	4.00	4.00
4.00	4.00	4.00	4.00	2.00	2.00	2.00	4.00	4.00
2.00	4.00	4.00	4.00	2.00	2.00	2.00	4.00	4.00
2.00	4.00	4.00	4.00	2.00	2.00	2.00	4.00	4.00
4.00	4.00	4.00	4.00	2.00	2.00	2.00	4.00	4.00
2.00	4.00	4.00	4.00	2.00	2.00	2.00	4.00	4.00
4.00	4.00	4.00	4.00	2.00	2.00	2.00	4.00	4.00
2.00	4.00	4.00	4.00	2.00	2.00	2.00	4.00	4.00
4.00	2.00	2.00	2.00	3.00	4.00	3.00	2.00	2.00
4.00	2.00	2.00	2.00	3.00	4.00	3.00	2.00	2.00
4.00	2.00	2.00	2.00	3.00	4.00	3.00	2.00	2.00
4.00	2.00	2.00	2.00	3.00	4.00	3.00	2.00	2.00
2.00	2.00	2.00	1.00	2.00	2.00	2.00	2.00	2.00
2.00	4.00	4.00	3.00	2.00	4.00	2.00	4.00	4.00
4.00	3.00	4.00	4.00	1.00	1.00	1.00	3.00	4.00
3.00	3.00	4.00	4.00	1.00	4.00	4.00	4.00	4.00
5.00	1.00	3.00	4.00	1.00	1.00	1.00	1.00	1.00
4.00	3.00	3.00	2.00	4.00	4.00	4.00	5.00	4.00
4.00	3.00	3.00	2.00	4.00	4.00	4.00	5.00	5.00
4.00	4.00	5.00	2.00	4.00	3.00	4.00	3.00	3.00
2.00	2.00	2.00	3.00	3.00	4.00	3.00	4.00	4.00
4.00	4.00	5.00	2.00	4.00	3.00	4.00	4.00	3.00
2.00	2.00	2.00	4.00	2.00	2.00	2.00	2.00	2.00
4.00	4.00	4.00	2.00	4.00	3.00	4.00	5.00	4.00
2.00	2.00	2.00	2.00	2.00	5.00	2.00	2.00	2.00
4.00	4.00	4.00	3.00	2.00	2.00	2.00	4.00	2.00
4.00	3.00	3.00	4.00	1.00	4.00	1.00	4.00	1.00

Effectivene	Effectivene	Effectivene	Economy1	Economy2	Economy3	Efficiency1	Efficiency2	Efficiency3
4.00	4.00	4.00	4.00	4.00	4.00	4.00	4.00	4.00
2.00	2.00	2.00	5.00	3.00	4.00	2.00	4.00	3.00
4.00	4.00	4.00	4.00	4.00	4.00	4.00	3.00	4.00
3.00	4.00	4.00	2.00	2.00	1.00	2.00	2.00	3.00
2.00	4.00	5.00	5.00	2.00	4.00	4.00	3.00	5.00
3.00	2.00	2.00	4.00	4.00	2.00	4.00	2.00	2.00
4.00	3.00	2.00	4.00	4.00	2.00	4.00	2.00	2.00
1.00	3.00	2.00	1.00	2.00	4.00	3.00	2.00	3.00
2.00	4.00	4.00	2.00	4.00	2.00	5.00	1.00	5.00
4.00	4.00	4.00	4.00	2.00	1.00	3.00	2.00	3.00
4.00	4.00	4.00	4.00	1.00	4.00	4.00	4.00	4.00
2.00	2.00	2.00	3.00	3.00	2.00	4.00	2.00	2.00
4.00	4.00	4.00	4.00	4.00	4.00	4.00	4.00	4.00
2.00	2.00	2.00	2.00	3.00	4.00	4.00	4.00	3.00
4.00	4.00	2.00	4.00	4.00	4.00	4.00	3.00	4.00
1.00	2.00	4.00	1.00	2.00	3.00	2.00	3.00	5.00
2.00	4.00	4.00	2.00	3.00	3.00	3.00	2.00	3.00
3.00	4.00	4.00	3.00	5.00	2.00	4.00	2.00	2.00
4.00	2.00	2.00	4.00	4.00	2.00	4.00	2.00	2.00
1.00	2.00	3.00	1.00	2.00	3.00	3.00	4.00	3.00
3.00	4.00	5.00	2.00	4.00	2.00	5.00	1.00	2.00
4.00	4.00	4.00	4.00	2.00	3.00	3.00	2.00	4.00
4.00	4.00	4.00	4.00	4.00	4.00	4.00	4.00	4.00
2.00	2.00	2.00	3.00	3.00	2.00	4.00	4.00	2.00
4.00	4.00	4.00	5.00	4.00	4.00	4.00	4.00	4.00
2.00	2.00	2.00	2.00	3.00	3.00	2.00	4.00	5.00
4.00	4.00	4.00	4.00	4.00	4.00	2.00	4.00	4.00
2.00	2.00	2.00	2.00	3.00	3.00	2.00	4.00	1.00
4.00	4.00	4.00	4.00	4.00	4.00	2.00	4.00	4.00
2.00	2.00	2.00	2.00	3.00	3.00	2.00	4.00	4.00
4.00	4.00	4.00	4.00	4.00	4.00	2.00	4.00	4.00
2.00	2.00	2.00	2.00	4.00	3.00	2.00	4.00	3.00
4.00	4.00	4.00	4.00	4.00	4.00	1.00	3.00	4.00
3.00	4.00	5.00	2.00	2.00	1.00	2.00	4.00	4.00
4.00	2.00	2.00	4.00	4.00	4.00	1.00	3.00	4.00
2.00	4.00	4.00	2.00	2.00	1.00	2.00	4.00	1.00
4.00	4.00	4.00	4.00	4.00	4.00	4.00	4.00	4.00
2.00	2.00	2.00	2.00	4.00	4.00	2.00	4.00	3.00
4.00	4.00	1.00	4.00	4.00	4.00	1.00	3.00	4.00
3.00	4.00	4.00	2.00	2.00	1.00	2.00	2.00	3.00
2.00	4.00	4.00	2.00	2.00	4.00	2.00	3.00	1.00
3.00	4.00	1.00	4.00	4.00	2.00	4.00	2.00	2.00
4.00	2.00	2.00	4.00	4.00	2.00	4.00	2.00	2.00
1.00	2.00	3.00	1.00	2.00	1.00	3.00	2.00	2.00
2.00	4.00	4.00	2.00	4.00	2.00	5.00	1.00	2.00
4.00	4.00	4.00	4.00	2.00	1.00	3.00	2.00	3.00

4.00	4.00	4.00	4.00	1.00	4.00	1.00	4.00	4.00
2.00	2.00	2.00	2.00	4.00	2.00	4.00	2.00	2.00
4.00	4.00	4.00	4.00	4.00	4.00	4.00	4.00	4.00
2.00	2.00	2.00	2.00	3.00	4.00	2.00	4.00	3.00
4.00	2.00	1.00	4.00	4.00	4.00	1.00	3.00	4.00
1.00	2.00	3.00	1.00	2.00	1.00	2.00	3.00	1.00
2.00	4.00	5.00	2.00	2.00	2.00	3.00	2.00	3.00
3.00	4.00	3.00	3.00	5.00	2.00	4.00	2.00	2.00
4.00	2.00	1.00	4.00	4.00	2.00	4.00	2.00	2.00
1.00	2.00	3.00	1.00	2.00	2.00	3.00	4.00	2.00
3.00	4.00	4.00	2.00	4.00	2.00	5.00	1.00	2.00
4.00	4.00	4.00	4.00	2.00	3.00	3.00	2.00	4.00
4.00	3.00	4.00	4.00	4.00	4.00	4.00	4.00	4.00
2.00	5.00	2.00	2.00	2.00	2.00	4.00	4.00	2.00
4.00	4.00	4.00	4.00	4.00	4.00	4.00	4.00	4.00
2.00	2.00	2.00	2.00	3.00	2.00	2.00	4.00	1.00
4.00	3.00	4.00	4.00	4.00	4.00	2.00	4.00	4.00
2.00	2.00	2.00	2.00	4.00	3.00	2.00	4.00	1.00
4.00	4.00	4.00	4.00	4.00	4.00	2.00	4.00	4.00
2.00	5.00	2.00	2.00	3.00	3.00	2.00	4.00	4.00
4.00	4.00	4.00	4.00	4.00	4.00	2.00	4.00	4.00
2.00	2.00	2.00	2.00	2.00	3.00	2.00	4.00	2.00
4.00	5.00	1.00	4.00	4.00	4.00	4.00	3.00	4.00
3.00	4.00	4.00	2.00	2.00	1.00	2.00	4.00	4.00
4.00	2.00	2.00	4.00	4.00	4.00	1.00	3.00	4.00
2.00	3.00	5.00	2.00	2.00	1.00	2.00	4.00	1.00
4.00	4.00	4.00	4.00	4.00	4.00	4.00	4.00	4.00
2.00	2.00	2.00	2.00	4.00	4.00	2.00	4.00	2.00
4.00	4.00	1.00	4.00	4.00	4.00	4.00	3.00	4.00
3.00	3.00	5.00	2.00	2.00	1.00	2.00	2.00	3.00
2.00	4.00	5.00	2.00	2.00	1.00	2.00	3.00	5.00
3.00	4.00	1.00	4.00	4.00	2.00	4.00	2.00	2.00
4.00	2.00	1.00	4.00	4.00	2.00	4.00	2.00	2.00
1.00	2.00	3.00	1.00	2.00	1.00	3.00	2.00	2.00
2.00	4.00	5.00	2.00	4.00	2.00	5.00	1.00	2.00
4.00	4.00	4.00	4.00	2.00	1.00	3.00	2.00	3.00
4.00	3.00	4.00	4.00	1.00	4.00	1.00	4.00	4.00
2.00	2.00	2.00	2.00	2.00	2.00	4.00	2.00	2.00
4.00	4.00	4.00	4.00	4.00	4.00	4.00	4.00	4.00
2.00	3.00	2.00	2.00	4.00	4.00	2.00	4.00	3.00
4.00	4.00	1.00	4.00	4.00	4.00	1.00	3.00	4.00

VAR00001	VAR00002	VAR00003	Performan	procureme	supplies_ma	supplies_sc	E-procurement
2.00	3.00	2.00	2.00	2.00	2.00	3.00	4.00
2.00	3.00	3.00	3.00	2.00	4.00	3.00	4.00
1.00	3.00	2.00	2.00	1.00	3.00	4.00	4.00
1.00	3.00	2.00	1.00	1.00	4.00	2.00	4.00
2.00	4.00	3.00	2.00	3.00	1.00	3.00	4.00
1.00	3.00	2.00	4.00	3.00	4.00	4.00	3.00
1.00	3.00	2.00	1.00	1.00	5.00	4.00	3.00
1.00	3.00	2.00	1.00	1.00	3.00	2.00	3.00
2.00	4.00	3.00	2.00	2.00	4.00	3.00	2.00
1.00	3.00	2.00	1.00	1.00	3.00	3.00	2.00
1.00	3.00	2.00	2.00	2.00	2.00	3.00	3.00
2.00	3.00	3.00	3.00	2.00	4.00	3.00	3.00
1.00	3.00	2.00	2.00	2.00	2.00	3.00	3.00
2.00	3.00	2.00	3.00	2.00	3.00	3.00	4.00
1.00	2.00	1.00	1.00	1.00	2.00	3.00	4.00
1.00	2.00	2.00	1.00	1.00	2.00	2.00	4.00
2.00	3.00	3.00	3.00	3.00	3.00	4.00	2.00
2.00	4.00	3.00	4.00	3.00	5.00	3.00	3.00
1.00	2.00	2.00	2.00	1.00	5.00	4.00	4.00
1.00	2.00	2.00	1.00	1.00	3.00	2.00	3.00
2.00	4.00	3.00	3.00	3.00	4.00	4.00	2.00
2.00	3.00	3.00	2.00	2.00	3.00	3.00	3.00
1.00	3.00	2.00	2.00	2.00	2.00	3.00	3.00
2.00	4.00	3.00	3.00	2.00	5.00	3.00	3.00
1.00	3.00	2.00	3.00	2.00	4.00	3.00	3.00
1.00	2.00	2.00	3.00	4.00	4.00	2.00	2.00
2.00	3.00	3.00	3.00	2.00	4.00	4.00	2.00
1.00	3.00	2.00	3.00	2.00	4.00	3.00	3.00
1.00	2.00	2.00	3.00	2.00	4.00	3.00	3.00
2.00	4.00	3.00	3.00	2.00	4.00	4.00	3.00
1.00	3.00	2.00	3.00	3.00	4.00	3.00	3.00
1.00	3.00	2.00	1.00	1.00	4.00	4.00	3.00
1.00	3.00	2.00	3.00	4.00	2.00	4.00	3.00
1.00	3.00	2.00	3.00	2.00	2.00	3.00	3.00
2.00	3.00	3.00	3.00	4.00	2.00	3.00	3.00
1.00	3.00	2.00	3.00	2.00	2.00	3.00	3.00
2.00	3.00	2.00	2.00	2.00	2.00	3.00	4.00
2.00	3.00	3.00	3.00	2.00	4.00	3.00	4.00
1.00	3.00	2.00	2.00	1.00	3.00	4.00	4.00
1.00	3.00	2.00	1.00	1.00	4.00	2.00	4.00
2.00	4.00	3.00	2.00	3.00	1.00	3.00	4.00
1.00	3.00	2.00	4.00	3.00	4.00	4.00	3.00
1.00	3.00	2.00	1.00	1.00	5.00	4.00	3.00
1.00	3.00	2.00	1.00	1.00	3.00	2.00	3.00
2.00	4.00	3.00	2.00	2.00	4.00	3.00	2.00
1.00	3.00	2.00	1.00	1.00	3.00	3.00	2.00

1.00	3.00	2.00	2.00	2.00	2.00	3.00	3.00
2.00	3.00	3.00	3.00	2.00	4.00	3.00	3.00
1.00	3.00	2.00	2.00	2.00	2.00	3.00	3.00
2.00	3.00	2.00	3.00	2.00	3.00	3.00	4.00
1.00	2.00	1.00	1.00	1.00	2.00	3.00	4.00
1.00	2.00	2.00	1.00	1.00	2.00	2.00	4.00
2.00	3.00	3.00	3.00	3.00	3.00	4.00	2.00
2.00	4.00	3.00	4.00	3.00	5.00	3.00	3.00
1.00	2.00	2.00	2.00	1.00	5.00	4.00	4.00
1.00	2.00	2.00	1.00	1.00	3.00	2.00	3.00
2.00	4.00	3.00	3.00	3.00	4.00	4.00	2.00
2.00	3.00	3.00	2.00	2.00	3.00	3.00	3.00
1.00	3.00	2.00	2.00	2.00	2.00	3.00	3.00
2.00	4.00	3.00	3.00	2.00	5.00	3.00	3.00
1.00	3.00	2.00	3.00	2.00	4.00	3.00	3.00
1.00	2.00	2.00	3.00	4.00	4.00	2.00	2.00
2.00	3.00	3.00	3.00	2.00	4.00	4.00	2.00
1.00	3.00	2.00	3.00	2.00	4.00	3.00	3.00
1.00	2.00	2.00	3.00	2.00	4.00	3.00	3.00
2.00	4.00	3.00	3.00	2.00	4.00	4.00	3.00
1.00	3.00	2.00	3.00	3.00	4.00	3.00	3.00
1.00	3.00	2.00	1.00	1.00	4.00	4.00	3.00
1.00	3.00	2.00	3.00	4.00	2.00	4.00	3.00
1.00	3.00	2.00	3.00	2.00	2.00	3.00	3.00
2.00	3.00	3.00	3.00	4.00	2.00	3.00	3.00
1.00	3.00	2.00	3.00	2.00	2.00	3.00	3.00
2.00	3.00	2.00	2.00	2.00	2.00	3.00	4.00
2.00	3.00	3.00	3.00	2.00	4.00	3.00	4.00
1.00	3.00	2.00	2.00	1.00	3.00	4.00	4.00
1.00	3.00	2.00	1.00	1.00	4.00	2.00	4.00
2.00	4.00	3.00	2.00	3.00	1.00	3.00	4.00
1.00	3.00	2.00	4.00	3.00	4.00	4.00	3.00
1.00	3.00	2.00	1.00	1.00	5.00	4.00	3.00
1.00	3.00	2.00	1.00	1.00	3.00	2.00	3.00
2.00	4.00	3.00	2.00	2.00	4.00	3.00	2.00
1.00	3.00	2.00	1.00	1.00	3.00	3.00	2.00
1.00	3.00	2.00	2.00	2.00	2.00	3.00	3.00
2.00	3.00	3.00	3.00	2.00	4.00	3.00	3.00
1.00	3.00	2.00	2.00	2.00	2.00	3.00	3.00
2.00	3.00	2.00	3.00	2.00	3.00	3.00	4.00
1.00	2.00	1.00	1.00	1.00	2.00	3.00	4.00
