# Supplementary material for: Procurement practices and value for money in State Corporations in Kenya
Source: PLoS One. 2024 Jun 18;19(6):e0303879. doi: 10.1371/journal.pone.0303879 (PMC11185499; doi:10.1371/journal.pone.0303879)
Supplement: Attachment — Submitted filename: Response to Reviewers.doc [file pone.0303879.s002.doc]

26/4/2024

Dear Editor

This is in response to comments made by reviewers to the manuscript entitled “Procurement Practices and Value for Money in State Corporations in Kenya” by John Muturi Waci, Peter Wang’ombe Kariuki and Purity Mukiri Mwirigi. We have gone through the comments and made revision as per the following Table.

Kindly note that we have updated the reference to include World Bank (2022), OECD, (2021); Lyra, et al.,(2022); Basheka, (2021). We have also removed from references OECD, 2015; OECD, 2017, World Bannk, 2012 and Lackert, 2009. This was to conform with reviewers comments.

We hope that you find our revised manuscript suitable for publication and look forward to hearing from you.

Sincerely,

John Muturi Waci

Business and economics,

University of Embu

6-60100 Embu, Kenya

Email: johnmuturi30@gmail.com

| **S/NO** | **COMMENT** | **IMPLEMENTATION** | **PAGE** |
| --- | --- | --- | --- |
| 1 | **Focus on Sampling:** While mentioning the study population is informative, the reviewers request a more detailed explanation of the sampling procedures used to select the state corporations involved in your research. Describe the specific methods used to ensure a representative sample. | I have described the procedure adopted which is stratified random sampling. The sampling procedure was used because the State Corporations in Kenya are categorized into eight sectors (strata) thus the method a representative sample from each sector or strata. The study also adopted a formula by Slovin’s to determine the appropriate sample size of 118 corporations to be used in the study. Table 1 indicates the population and the sample size for each category. | 12 |
| 2 | **East African Focus:** Within the revised literature review, incorporate studies specifically from the East African region, including Kenya, Uganda, Tanzania, and others. | Studies from East African and other African countries have been included in the literature review i.e. Mchopa (2015) on VFM in Tanzania, Basheka (2021) on public procurement governance in Uganda, Onyango (2014) on procurement planning in Kenya; Dede and Theuri (2018) on supplies sourcing in Kenya; Botlhale (2017) on VFM in Botswana, Asare and Prempeh (2016) examined VFM in Ghana; and many more. | 8-11 |
| 3 | **Recent References:** Update references throughout the literature review to ensure reliance on the most current sources. | Updated the document to include current sources |  |
| 4 | **Presentation Improvement:** Enhance the presentation of the methodology section by referring to top journals and articles in the fields of business and social science research. | Enhanced the methodology by citing previous similar studies in Table 2 | 14 |
| 5 | **Section Renaming/Merging:** Reviewer 2 suggests considering renaming or merging certain sections, particularly within the introduction, to enhance clarity and flow. Recheck references within the introduction for accuracy and consistency. | Merged theoretical literature section and literature review and hypothesis to one section known us “Literature Review and Hypotheses Development” | 6 |
| 5 | **Abstract Revisions:** Move recommendations from the abstract to a separate paragraph within the conclusion section. Highlight the originality of your research within the abstract. | Moved the recommendation to conclusion and highlighted the originality of the research. | 1 & 24 |
| 6 | **Comparative Analysis:** Compare your findings with results from previous similar studies in the domain of procurement practices and value for money. | Compared the findings with results from previous studies. | 22-23 |

**REVIEWERS COMMENTS AND IMPLEMENTATION**
